# Regulation of Butyrate-Induced Resistance through AMPK Signaling Pathway in Human Colon Cancer Cells

**DOI:** 10.3390/biomedicines9111604

**Published:** 2021-11-03

**Authors:** Hee Young Yoo, So Yeon Park, Sun-Young Chang, So Hee Kim

**Affiliations:** College of Pharmacy and Research Institute of Pharmaceutical Science and Technology, Ajou University, Suwon 16499, Korea; yoo1454@ajou.ac.kr (H.Y.Y.); qkrth0413@ajou.ac.kr (S.Y.P.); sychang@ajou.ac.kr (S.-Y.C.)

**Keywords:** butyrate resistance, colon cancer, AMPK, autophagy, ACC, Akt, mTOR

## Abstract

Butyrates inhibit cell growth in colon cancer cells by inhibiting histone deacetylases. However, chronic exposure to butyrates induces butyrate resistance in colon cancer cells. The mechanism underlying the acquisition of resistance is not yet fully understood. Here, butyrate-resistant (BR) colon cancer cells were developed in HCT116, HT29, and SW480 human colon cancer cells and were confirmed by the increase in the inhibitory concentrations of cell growth by 50% (IC_50_) compared to their respective parental (PT) cells. Chronic exposure to butyrate induced autophagy via higher expression of Beclin-1 and LC3B-II. The AMP-activated protein kinase (AMPK) was downregulated along with the activation of Akt and mammalian target of rapamycin (mTOR) and decrease in acetyl-CoA carboxylase (ACC) in BR colon cancer cells compared to those in their respective PT cells. Activation of AMPK by AICAR treatment in BR colon cancer cells suppressed cell proliferation by inhibiting Akt and mTOR and activating ACC. Taken together, chronic exposure to butyrate increased butyrate resistance in human colon cancer by inducing protective autophagy through the downregulation of AMPK/ACC and activation of Akt/mTOR signaling. Activation of AMPK restored sensitivity to butyrate by the inhibition of Akt/mTOR, suggesting that AMPK could be a therapeutic target for BR colon cancers.

## 1. Introduction

AMP-activated protein kinase (AMPK) is a key regulator for balancing energy supply and maintaining homeostasis and ultimately protects cells from stressful conditions by rearranging multiple metabolic pathways [1]. The activation of AMPK inhibits the proliferation of cancer cells by increasing the expression of p21, p27, and p53 and inhibiting phosphorylation of the Akt/mammalian target of rapamycin (mTOR) signaling pathway [2,3]. In particular, the activation of AMPK has recently been shown to coordinate metabolic reprogramming in drug-resistant cancer cells, including the promotion of the Warburg effect and induction of mitochondrial biosynthesis [4,5,6]. Furthermore, phosphorylation of AMPK is important for mediating the induction of autophagy, a process that is effective in modulating and restoring chemosensitivity by the breakdown of cellular components to meet nutrient requirements under harmful stresses [5].

Autophagy is an essential homeostatic, programmed catabolic process that is activated by various stimuli and is important for the breakdown or recycling of proteins and cellular components [7,8]. Dysregulation of autophagy is associated with tumorigenesis and resistance to cancer therapy [9,10]. A controlled increase in autophagy contributed to improved cell viability. However, overactivation of autophagy accelerates apoptosis [8,11]. Autophagy is believed to be crucial in the process of drug resistance in various cancer cells, such as glioma, osteosarcoma, and acute myeloid leukemia [9]. Autophagy has emerged as a potential mechanism associated with acquired resistance to anti-epidermal growth factor receptor (EGFR) therapy. Anti-EGFR treatment may induce autophagy, eventually resulting in resistance to anti-EGFR therapy [12,13]. Thus, targeting autophagy can be one of the approaches to overcome resistance to anti-cancer drugs as well as anti-EGFR therapy.

Butyrate is a major short-chain fatty acid produced from dietary fiber through fermentation by intestinal microbes in normal colon cells and is further oxidized to acetyl-CoA in the mitochondria for use as an energy source for normal colon cells; however, in colon cancer cells, butyrate is a histone deacetylase inhibitor. It acts as a cell growth inhibitory agent and induces differentiation [14,15,16]. However, when colon cancer cells are continuously exposed to butyrate, anticancer activity is lost as the cells become resistant to butyrate [17,18] and eventually become resistant to butyrate [18]. BCS-TC2 human colon adenocarcinoma cells [19] and HeLa human cervical cancer cells [20] also showed resistance to butyrate.

In this study, the effect of butyrate resistance on autophagy in colon cancer cells was evaluated. Changes in the AMPK signaling pathway as a mechanism of butyrate resistance in butyrate-resistant (BR) colon cancer cells were examined, and the effect on the fatty acid metabolism was also investigated during the acquisition of butyrate resistance.

## 2. Materials and Methods

### 2.1. Materials

Fetal bovine serum (FBS), Dulbecco’s modified Eagle medium DMEM, and Dulbecco’s phosphate-buffered saline (DPBS) were obtained from Invitrogen (Carlsbad, CA, USA). Penicillin-streptomycin was purchased from GenDEPOT (Katy, TX, USA). Sodium butyrate, 3-(4,5-dimethylthiazol-2-yl)-2,5-diphenyl-tetrazolium-bromide (MTT), and 5-aminoimidazole-4-carboxamide ribonucleotide (AICAR) were supplied by Sigma-Aldrich (St. Louis, MO, USA). Antibodies against p21; cyclin A; cyclin D1; cyclin E; Bad; acetyl-CoA carboxylase (ACC); ATP citrate lyase (ACLY); fatty acid synthase (FASN); LC3B; Beclin-1; p62; phospho-AMPKα (Thr172); AMPKα; mammalian target of rapamycin (mTOR); phospho-mTOR (Ser2448); Akt; phospho-Akt (Ser473); phospho-ACC (Ser79); carnitine palmitoyltransferase 1A (CPT1); breast cancer resistance protein (BCRP); Bax; P-glycoprotein (P-gp); and poly ADP ribose polymerase (PARP) were purchased from Cell Signaling Biotechnology (Beverly, MA, USA). Actin, short-chain acyl-CoA dehydrogenase (SCAD), and p53 antibodies were obtained from Santa Cruz Biotechnology (Santa Cruz, CA, USA). Long-chain acyl-CoA dehydrogenase (LCAD) antibody, propidium iodide, and RNase A were purchased from Abcam (Cambridge, UK). All other chemicals and reagents were of analytical or HPLC grade and were used without further purification.

### 2.2. Cell Culture and Establishment of Butyrate-Resistant Colon Cancer Cells

Parental HCT116, HT29, and SW480 human colon cancer cells were purchased from Koran Cell Line Bank (KCLB, Seoul, Korea) and were cultured in DMEM with 10% FBS, 100 U/mL penicillin, and 10 mg/mL streptomycin at 37 °C in a humidified 5% CO_2_ incubator. To establish BR colon cancer cells (HCT116/BR, HT29/BR, and SW480/BR), a previously published method was used [18]. The butyrate stock solution was prepared by dissolving sodium butyrate in DPBS. Cells were incubated overnight to allow cell attachment before the butyrate treatment was applied. Parenteral (PT) colon cancer cells of HCT116, HT29, and SW480 (HCT116/PT, HT29/PT, and SW480/PT) were initially incubated with serum-containing media supplemented with 0.2 mM sodium butyrate. The initial butyrate treatment induced the death of most cells; some cells survived but proliferated slower compared to their respective PT colon cancer cells. When the cells were 80% confluent, they were cultured in the next passage with fresh media containing the same concentration of butyrate. By continuously growing cells in the presence of the same concentration of butyrate until no more detectable death of cells was observed, the concentration of butyrate was increased by 0.2 mM. After culturing cells with a step-wise increase in butyrate concentration to a maximum of 1.6 mM over approximately 3–6 months, BR colon cancer cells were established. For comparison, acute treatment (AT) with butyrate at 1.6 or 6.4 mM (AT1.6 or AT6.4) was also applied to PT colon cancer cells of HCT116, HT29, and SW480 (HCT116/AT1.6 or 6.4, HT29/AT1.6 or 6.4, and SW480/AT1.6 or 6.4) for 24 h and was confirmed the concentration-dependency of butyrate on the cell cycle arrest and autophagy.

### 2.3. Cell Proliferation Assay

To determine the effect of butyrate-resistance and chemoresistance on the proliferation of colon cancer cells, HCT116/PT, HT29/PT, and SW480/PT colon cancer cells and their respective BR cells were seeded onto 96-well plates. Cells were treated with various concentrations of butyrate, doxorubicin, 5-fluorouracil (5-FU), and oxaliplatin. After 72 h incubation, the medium was removed, diluted MTT solution (100 μL) was added, and the plate was incubated at 37 °C for an additional 2 h. Subsequently, the purple formazan crystals were solubilized by adding dimethyl sulfoxide (DMSO), and the absorbance was read at 540 nm by using an ELISA reader (Bio-Tek Instruments Inc., Winooski, VT, USA).

In order to investigate the effects of AMPK activation on cell proliferation in BR colon cancer cells, HCT116/BR, HT29/BR, and SW480/BR cells were treated with DPBS, 0.2 mM AICAR, 3.2 mM butyrate, and 0.2 mM AICAR for 1 h followed by 3.2 mM butyrate for 72 h. Other procedures were the same as described above.

### 2.4. Flow Cytometry

In order to assess cell cycle progression, each PT colon cancer cell line and its respective BR colon cancer cells were plated onto 60 mm dishes. After overnight incubation, PT and BR colon cancer cells were treated with DPBS and 1.6 mM butyrate for 24 h. Cells were then trypsinized, centrifuged for 5 min at 500× *g* at 4 °C, fixed with 70% ethanol at 4 °C, and 1 mL of propidium iodide solution (final concentration, 50 μg/mL) containing 100 U/mL of RNase A and 0.1% glucose was added for 30 min in the dark [18,21]. Flow cytometry was then performed by using a FACSCalibur flow cytometer (FACSDiva7.0, Becton-Dickinson, San Jose, CA, USA), with cells identified using the Cell Quest software (Becton-Dickinson). Red fluorescence, indicative of propidium iodide uptake by damaged cells, was measured at 585/542 nm by logarithmic amplification and electronic compensation for spectral overlap [18,21].

### 2.5. Immunofluorescence Analysis

PT, AT1.6, and BR colon cancer cells were fixed with 1.6 mM butyrate for 24 h and fixed with 4% paraformaldehyde (pH 7.4) at room temperature for 10 min and permeabilized with 0.1% Triton X-100 (Sigma-Aldrich) for 10 min at room temperature. Cells were washed with DPBS three times and incubated overnight at 4 °C with an LC3B antibody diluted at 1:50. After washing three times with DPBS, the cells were incubated with fluorescence-conjugated secondary antibody (Alexa Fluor 488, Invitrogen), diluted at 1:100 at room temperature for 1 h, and washed three times with DPBS. The cells were carefully mounted with a coverslip by using a mounting medium containing DAPI (Sigma-Aldrich). Fluorescence images were analyzed by using a confocal microscope (Nikon, Minato City, Japan).

### 2.6. RNA Extraction and Reverse Transcription

Total RNA was isolated from PT, AT1.6, and BR colon cancer cells using Trizol (Invitrogen), followed by column purification using the RNeasy Mini kit (Qiagen, Valencia, CA, USA) according to the manufacturer’s instructions. RNA was eluted from the spin column using RNase-free dH_2_O. Complementary DNA (cDNA) was prepared from RNA samples by using the High Capacity cDNA Reverse Transcription kit (Applied Biosystems, Foster City, CA, USA) according to the manufacturer’s protocols.

### 2.7. Quantitative Real-Time Reverse Transcription PCR

Quantitative real-time reverse transcription PCR (qRT-PCR) was performed using Power SYBR Green Master Mix (Applied Biosystems), and qRT-PCR was performed using the StepOne Real-Time PCR System (Applied Biosystems). Each sample final volume was 20 μL, containing approximately 100 ng of cDNA. The sequences of the oligonucleotide primers for *CPT1A*, *LCAD*, *SCAD*, *ACC*, *ACLY*, *FASN*, and glyceraldehyde 3-phosphate dehydrogenase (*GAPDH*) are listed in Table 1. *GAPDH* was used to standardize the qRT-PCR results for *CPT1A*, *LCAD*, *SCAD*, *ACC*, *ACLY*, and *FASN*. The relative mRNA levels were estimated by using the 2^−^^ΔΔCt^ method described previously [22,23].

### 2.8. Protein Isolation and Immunoblot Analysis

In order to evaluate protein expressions, PT, BR, AT1.6, and/or AT6.4 colon cancer cells were washed three times with cold DPBS, and the cells were harvested with lysis buffer supplemented with protease inhibitor cocktail (Sigma-Aldrich). Proteins were quantified by using a BCA assay kit (Pierce, Rockford, IL, USA) according to the manufacturer’s protocol. To investigate the effects of AMPK activation in BR colon cancer cells, BR cells were treated with DPBS or 1 mM AICAR for 24 h. Other procedures were similar to those described above. Proteins (20–40 μg) were quantified using a BCA assay kit (Pierce, Rockford, IL, USA) according to the manufacturer’s protocol. Proteins (20–40 μg) were resolved on 7.5–15% SDS-PAGE and transferred to nitrocellulose membranes (Pall Corp., Ann Arbor, MI, USA). The blots were incubated with primary antibody diluted at 1:1000 or actin diluted at 1:10,000. Actin was used as the loading control.

### 2.9. Statistical Analysis

Data are presented as means ± standard deviation (SD). The significance of differences was estimated using Tukey’s *post-test* for comparison among three means or more after analysis of variance (ANOVA) or Student’s *t*-test between two unpaired data using GraphPad Prism 5.0 software (GraphPad, La Jolla, CA, USA). Statistical significance was set at *p* < 0.05.

## 3. Results

### 3.1. Resistance to the Cell Proliferation of BR Colon Cancer Cells

**Figure 1:**
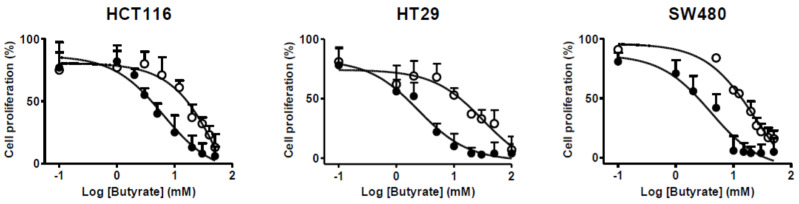
Effects of sodium butyrate on the cell proliferation in colon cancer cells. Cell proliferation was evaluated with an MTT assay in parental (PT, ●) and butyrate-resistant (BR, ○). HCT116 and SW480 cells were incubated with 0–50 mM of sodium butyrate for 72 h. HT29 cells were incubated with 0–100 mM of sodium for 72 h. The data are expressed as mean ± standard deviation of triplicate experiments.

Butyrate resistance was induced in HCT116, HT29, and SW480 human colon cancer cells by chronic exposure to butyrate for approximately six months. Generally, cell morphology was altered slightly, and the growth rates of the BR colon cancer cells were slower than those of their respective PT colon cancer cells. Cell proliferation was inhibited by butyrate in a concentration-dependent manner in both PT and BR colon cancer cells (Figure 1). The inhibitory concentrations of cell growth by 50% (IC_50_) values of butyrate in HCT116/BR, HT29/BR, and SW480/BR cells were significantly increased by 15.2, 14.0, and 6.08-fold, respectively, compared to those of their respective PT cells (Table 2), confirming that resistance to butyrate was induced in BR colon cancer cells. 

To examine whether butyrate resistance induced chemoresistance, both PT and BR colon cancer cells of HCT116, HT29, and SW480 were treated with doxorubicin, 5-FU, and oxaliplatin at various concentrations for 72 h. The IC_50_ values are shown in Table 2. The inhibition of cell proliferation increased in a concentration-dependent manner in both PT and BR colon cancer cells, but BR colon cancer cells exhibited greater chemoresistance than the respective PT colon cancer cells. The IC_50_ values in BR colon cancer cells for oxaliplatin, doxorubicin, and 5-FU were approximately 4.75–8.00, 3.41–4.62, and 2.91–3.64, respectively, than those of their respective PT colon cancer cells.

### 3.2. Effects of Butyrate Resistance on Drug Efflux Pumps

**Figure 2:**
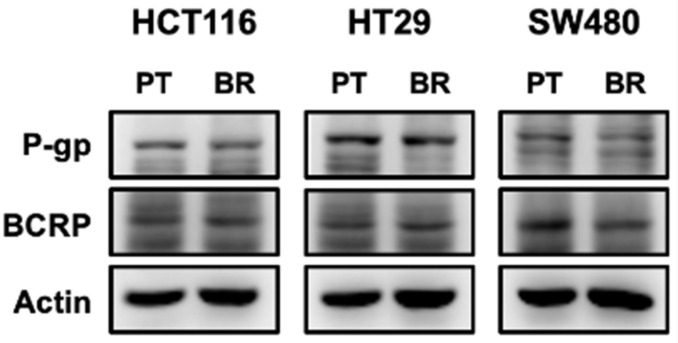
Effects of butyrate resistance on drug efflux pump expression. Expression of P-glycoprotein (P-gp) and breast cancer resistance protein (BCRP) was determined by immunoblot analysis. Actin was used as an internal control. This was repeated in three independent experiments.

Drug efflux pumps are associated with drug resistance. In particular, ATP-binding cassette (ABC) transporters are proteins that efflux the absorbed drugs from the small intestinal epithelial cells and are considered as one of the possible mechanisms of butyrate resistance. Therefore, we determined P-gp, BCRP, and MRP1 protein levels by immunoblotting (Figure 2). There was no significant difference in the expression of P-gp and BCRP in either PT or BR colon cancer cells, and MRP1 was not detected in either PT or BR cells. Therefore, drug efflux pumps do not appear to affect butyrate and anticancer drug resistance in BR colon cancer cells.

### 3.3. Effects of Butyrate Resistance on the Cell Cycle Progression

**Figure 3:**
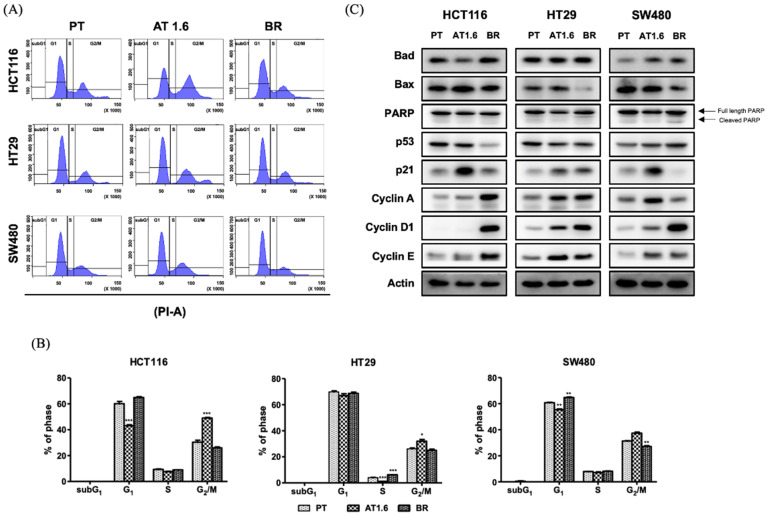
Effects of butyrate resistance on the cell cycle progression in parental (PT), 1.6 mM sodium butyrate-treated PT for 24 h (AT1.6), and butyrate-resistant (BR) colon cancer cells of HCT116, HT29, and SW480. (**A**) Cellular DNA was analyzed by flow cytometry. (**B**) The number of cells in each phase was calculated from each histogram. (**C**) Protein expression was analyzed by immunoblot analysis. Actin was used as an internal control. The data are expressed as mean ± standard deviation of triplicate experiments. The statistics showed only the comparison with PT cells. * *p* < 0.05; ** *p* < 0.01; *** *p* < 0.001.

Butyrate induces cell cycle arrest and apoptosis in cancer cells [24]; therefore, we performed flow cytometry to assess the effect of butyrate on cell cycle progression in PT and BR colon cancer cells. We also treated PT colon cancer cells with 1.6 mM butyrate for 24 h (HCT116/AT1.6, HT29/AT1.6, and SW480/AT1.6) and compared PT and BR colon cancer cells. As shown in Figure 3A,B, the patterns of cell cycle progression among the three BR colon cancer cells were similar, and G_1_ and S phases were not significantly different compared to those in their respective PT colon cancer cells. However, the proportion of cells in the G_2_/M phase was significantly increased by 53.6%, 22.1%, and 19.4% in HCT116/AT1.6, HT29/AT1.6, and SW480/AT1.6 cells, respectively, compared to that in their respective PT colon cancer cells, but it was restored to the basal levels of PT colon cancer cells in BR colon cancer cells. No subG_1_ peaks were observed in PT, AT1.6, and BR colon cancer cells, indicating that no apoptosis took place in PT, AT1.6, and BR colon cancer cells.

Immunoblot analysis supported these results (Figure 3C). The expression of cyclin D1 and cyclin E significantly increased in all BR colon cancer cells compared to that in their respective PT colon cancer cells. Cyclin A expression greatly increased in the BR colon cancer cells of HCT116 and HT29, but no changes were observed in SW480/BR cells compared to SW480/PT cells. Expression of p21 greatly increased in all AT1.6 colon cancer cells, but the increase in the expression level of p21 was markedly lower in BR cells compared to that in their respective AT1.6 cells or returned to the level of PT cells (Figure 3C). The expression of p53 decreased in HCT116/BR cells, but there was no significant change in AT1.6 and other BR colon cancer. The expression of Bax, a proapoptotic protein, was increased in HCT116/AT1.6 colon cancer cells but decreased to the level of their respective PT colon cancer cells of HT29 and SW480 cells. The changes in cleaved PARP were minimal in AT1.6 and BR colon cancer cells, consistent with the results that no subG_1_ peak was observed.

### 3.4. Effect of Butyrate Resistance on the Induction of Autophagy

**Figure 4:**
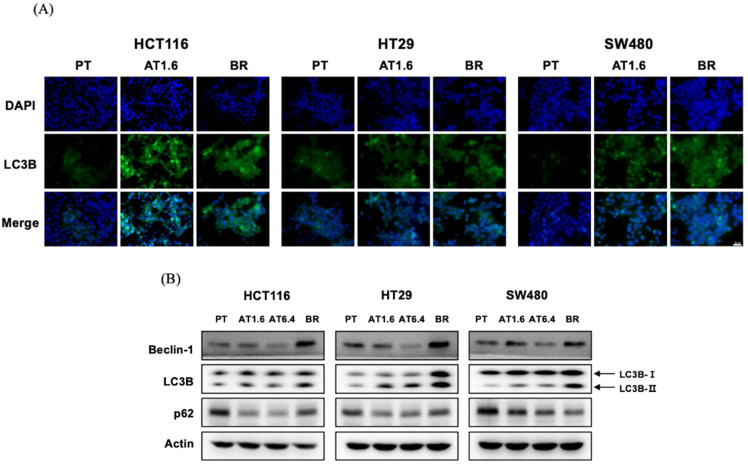
Effects of butyrate resistance on the induction of autophagy in parental (PT), 1.6 and/or 6.4 mM sodium butyrate-treated PT for 24 h (AT1.6 and/or AT6.4), and butyrate-resistant (BR) colon cancer cells of HCT116, HT29, and SW480. (**A**) Fluorescence was visualized by confocal microscopy. LC3B protein was labeled green (Alexa 488), and the nucleus was stained with DAPI. The size of scale bar was 100 µm. (**B**) Protein expression of Beclin-1, LC3B, and p62 was determined by immunoblot analysis. Actin was used as an internal control. This was repeated in three independent experiments.

In order to determine the effects of butyrate resistance on the induction of autophagy, the expression of LC3B, an important constituent of the autophagosomal membrane [25], was analyzed by using immunofluorescent staining to visualize the expression of LC3B (Figure 4A). LC3B-II expression increased in both AT1.6 and BR colon cancer cells compared to that in their respective PT colon cancer cells, indicating that autophagy was induced by acute and chronic exposure to butyrate. The induction of autophagy was confirmed by immunoblot analysis of LC3B (Figure 4B). The expression of LC3B-II increased in AT1.6 and AT6.4 cells and was much greater in BR colon cancer cells than that in the respective PT cells (Figure 4B). In addition, the expression of Beclin-1, which is involved in the initiation phase of autophagy [25], also increased in BR colon cancer cells compared to that in their respective PT, AT1.6, and AT6.4 colon cancer cells. However, p62 level decreased in AT1.6, AT6.4, and BR colon cancer cells compared to that in their respective PT cells, indicating that lysosomal degradation of autophagosomes results in a decrease in p62 levels during autophagy [26,27]. No dependency on butyrate concentration was observed in AT1.6 and AT6.4 cells.

### 3.5. Effects of Butyrate Resistance on the AMPK Signaling Pathway

**Figure 5:**
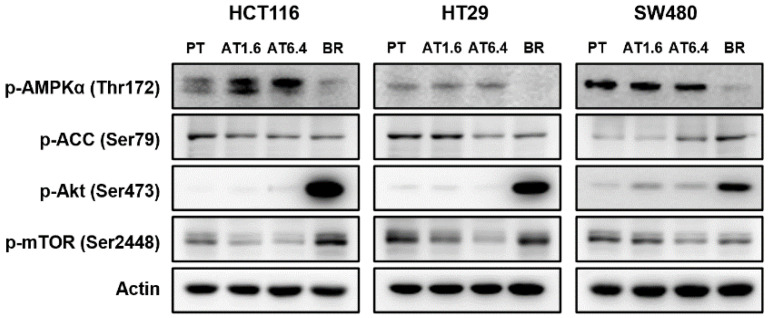
Effects of butyrate resistance on AMPK/ACC and Akt/mTOR signaling in the parental (PT), 1.6 and/or 6.4 mM sodium butyrate-treated PT for 24 h (AT1.6 and/or AT6.4) and butyrate-resistant (BR) colon cancer cells of HCT116, HT29, and SW480. The expression of phospho-AMPKα (Thr172), phospho-ACC (Ser79), phospho-Akt (Ser473), and phospho-mTOR (Ser2448) proteins was determined by immunoblot analysis. Actin was used as an internal control. This was repeated in three independent experiments. p-, phospho-.

In order to explore whether the autophagy induced by chronic exposure with respect to butyrate was related to AMPK/ACC and Akt/mTOR signaling pathways, immunoblot analysis was performed in PT, AT1.6, AT6.4, and BR colon cancer cells of HCT116, HT29, and SW480 (Figure 5). The protein levels of phospho-AMPKα (Thr172) were downregulated in BR colon cancer cells compared to those in PT, AT1.6, and AT6.4 cells of HCT116, HT29, and SW480. The expression of phospho-ACC, a downstream molecule of AMPK, was inhibited in the BR colon cancer cells of HCT116 and HT29 compared to that in their respective PT cells. Phospho-Akt (Ser473), an upstream of AMPK, was greatly overexpressed in BR colon cancer cells compared to those in their respective PT, AT1.6, and AT6.4 cells. The levels of phospho-mTOR (Ser2448), a downstream target of both AMPK and Akt, decreased in AT1.6 and AT6.4 cells but increased in BR colon cancer cells compared to that of their respective PT cells or returned to the basal level of PT cells in BR colon cancer cells. Again, no butyrate concentration dependency was observed in AT1.6 and AT6.4 cells.

### 3.6. Activation of AMPK in BR Colon Cancer Cells

**Figure 6:**
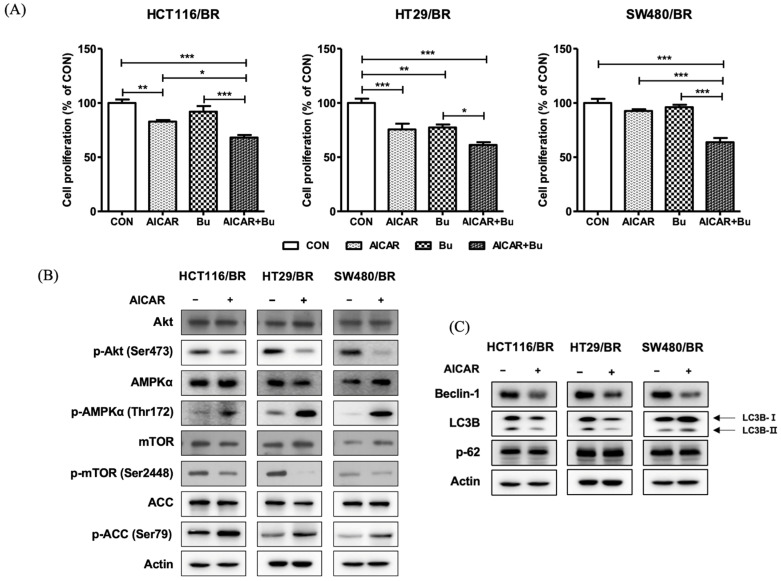
Activation of AMPK in butyrate-resistant (BR) colon cancer cells of HCT116, HT29, and SW480 by treatment with AICAR. (**A**) BR colon cancer cells were pretreated with or without 0.2 mM AICAR for 1 h, followed by 3.2 mM sodium butyrate for 72 h. Cell proliferation was determined by an MTT assay. (**B**,**C**) BR colon cancer cells were treated without or with 1 mM AICAR for 24 h. Protein expression was determined by immunoblot analysis. Actin was used as an internal control. Data are expressed as mean ± standard deviation of triplicate experiments. CON, control; Bu, Butyrate; AICAR, 5-aminoimidazole-4- carboxamide ribonucleotide; p-, phosphor-; * *p* < 0.05; ** *p* < 0.01; *** *p* < 0.001.

In order to examine the effect of increasing AMPK activity in BR colon cancer cells, BR cells were treated with 0.2 mM AICAR, a well-known AMPK activator [2], for 1 h, then treated with 3.2 mM butyrate for 72 h, and the inhibitory effect on cell proliferation was observed (Figure 6A). When treated with AICAR alone, the inhibition of cell proliferation significantly increased in HCT116/BR and HT29/BR cells compared to that in their respective untreated BR cells. When treated with both butyrate and AICAR, the inhibition of cell proliferation significantly increased in all BR colon cancer cells compared to those in BR treated with AICAR or butyrate alone, suggesting that activation of AMPK by AICAR significantly increased the inhibitory effect on BR colon cancer cell proliferation. 

Protein expression of the AMPK signaling pathway demonstrated that phospho-AMPKα (Thr172) and phospho-ACC (Ser79) levels significantly increased, whereas phospho-Akt (Ser473) and phospho-mTOR (Ser2448) levels decreased in BR colon cancer cells of HCT116, HT29, and SW480 treated with AICAR (Figure 6B). The expression of Beclin-1 decreased in all BR cells of HCT116, HT29, and SW480, and LC3B-II also decreased in HCT116/BR and HT29/BR cells but slightly increased in SW480/BR cells. The expressions of p62 were comparable in all three BR cells regardless of AICAR treatment (Figure 6C).

### 3.7. Effects of Butyrate Resistance on Enzymes Involved in the Fatty Acid Metabolism

**Figure 7:**
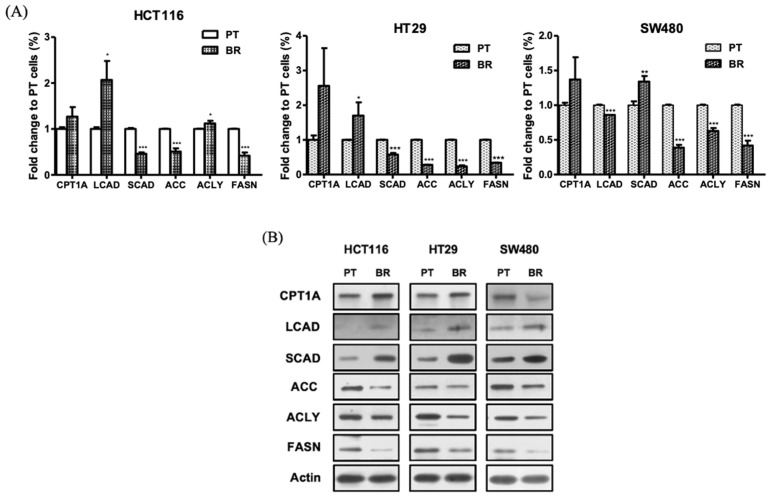
Effects of butyrate resistance on enzymes involved in fatty acids metabolism in the parental (PT) and butyrate-resistant (BR) colon cancer cells of HCT116, HT29, and SW480. (**A**) Gene expression of *CPT1A, LCAD*, *SCAD,* *ACLY*, *ACC,* and *FASN* was analyzed by quantitative real-time reverse transcription-PCR (qRT-PCR). (**B**) Protein expression of CPT1A, LCAD, SCAD, ACLY, ACC, and FASN was analyzed by immunoblot analysis. Actin was used as an internal control. Data are expressed as mean ± standard deviation of triplicate experiments. * *p* < 0.05; ** *p* < 0.01; *** *p* < 0.001.

In order to investigate whether the decrease in ACC in BR colon cancer cells is related to fatty acid metabolism, the enzymes involved in fatty acid catabolism and synthesis were determined by qRT-PCR and immunoblot analyses. The gene and protein expression of enzymes involved in fatty acid catabolism, such as CPT1A, LCAD, and SCAD, did not appear to be consistent in both PT and BR colon cancer cells, especially SCAD. The gene expression of SCAD in BR colon cancer cells was significantly lower but protein expressions of SCAD were significantly higher than compared to their respective PT cells. On the contrary, gene and protein expression of enzymes involved in the fatty acid synthesis, such as ACLY, ACC, and FASN, significantly decreased (Figure 7), which is assumed that acetyl-CoA produced during fatty acid metabolism or glycolysis can be utilized as an energy source for the growth of BR colon cancer cells rather than for fatty acid synthesis.

## 4. Discussion

Dietary fiber consumed as food is fermented by intestinal microflora and broken down into short-chain fatty acids, including butyrates that inhibit tumor cell growth and enhance differentiation while inducing glutathione S-transferase (GST), increasing anticancer drug resistance [14]. We previously reported that the early inhibitory effect of HCT116 colon cancer cell proliferation disappeared when HCT116 colon cancer cells were chronically exposed to butyrate, indicating that resistance to butyrate was developed in HCT116 colon cancer cells. Butyrate resistance also induces chemoresistance by increasing IC_50_ values, resulting in poor responses to anticancer drugs, such as cell motility, apoptosis, and invasion [18].

The significant effects on cell proliferation and G_2_ arrest shown in AT1.6 and/or AT6.4 disappeared gradually by chronic exposure of colon cancer cells to butyrate, resulting in the restoration of G_2_ arrest in BR cells to that observed in untreated PT cells, confirming the development of resistance to butyrate. This may occur because the drug is not taken up by cells due to an increase in efflux pumps, such as P-gp, MRP1, or BCRP. However, the expression of P-gp and BCRP in BR colon cancer cells was comparable to that in PT cells, indicating that P-gp and BCRP were not involved in butyrate resistance. The relationship between butyrate resistance and P-gp and/or BCRP levels seems to be controversial. It has been reported that butyrate increases the expression of P-gp through upregulation of signal transducer and activator of transcription 3 (STAT3) and mRNA stabilization of *MDR1* [28]. In contrast, the expression and function of intestinal P-gp decreased, but those of intestinal BCRP increased in rats treated with short-chain fatty acids [29].

As shown in Figure 3, butyrate resistance in BR colon cancer cells might be caused by the increased expression of tumorigenic proteins, such as cyclin A, cyclin D1, and cyclin E, or decreased expression of pro-apoptotic proteins, such as Bax, p21, p53, and Bad. This is contrary to the increased expression of pro-apoptotic proteins and decreased expression of tumorigenic proteins in AT1.6 cells. Therefore, butyrate resistance might be attributable to the changes in the expression of tumorigenic and/or pro-apoptotic proteins rather than changes in the expression of P-gp or BCRP, which were found comparable between PT and BR colon cancer cells. The Bcl-2 family is also involved in butyrate resistance. Bcl-xL levels significantly increased, but Bim and Bax levels decreased in HCT116/BR cells [18]. Similar results were also reported in BCS-TC2 human colon adenocarcinoma cells, where resistance to butyrate induces the impairment of the mitochondrial apoptosis pathway through inactivation of Bax and upregulation of Bcl-2 [19]. Activation of Bcl-2 is also involved in protective autophagy in cancer stem cells via EGFR signaling [30,31]. HeLa human cervical cancer cells also show resistance to butyrate via upregulation of cyclin D1 [20].

Contrary to the results of cell proliferation and G_2_ cell cycle arrest, autophagy was observed via the upregulation of LC3B-II in both AT1.6 and BR colon cancer cells. It was observed that the expression of Beclin-1 and LC3B-II proteins was higher in BR colon cancer cells than that in AT1.6 cells. In AT1.6 and AT6.4 colon cancer cells, treatment with butyrate inhibited cancer cell proliferation by an increase in phospho-AMPKα expression followed by a decrease in phospho-mTOR, resulting in the tumor-suppressive autophagy in AT1.6 colon cancer cells. On the other hand, it is thought that autophagy in BR colon cancer cells protects and supports BR colon cancer cells via inducing excessive activation of Akt and inhibition of AMPK phosphorylation and resulting in the induction of mTOR phosphorylation. This seems to use energy efficiently by removing unnecessary proteins from cancer cells for the survival of cancer cells that, thus, become resistant to butyrate or anticancer drugs [4,5,32].

Similar results have been reported for several anti-cancer therapies. Under normal conditions, phosphoinositide 3-kinase (PI3K)/Akt/mTOR signaling inhibits autophagy through the activation of EGFR, but autophagy is induced for cellular survival under stress conditions. Anti-EGFR therapies, such as gefitinib, erlotinib, and cetuximab, promote autophagy in non-small cell lung cancer as a survival mechanism and result in resistance to anti-EGFR therapy [12,33]. Protective autophagy also causes resistance to erlotinib in head and neck squamous cell carcinomas [34]. Chronic exposure to sorafenib causes acquired resistance to sorafenib in human hepatocellular carcinoma (HCC) cells [35]. Sorafenib induces tumor-suppressive autophagy in the parental HCC cells but induces the phosphorylation of Akt followed by mTOR and causes protective autophagy in sorafenib-resistant HCC cells [35]. Therefore, targeting autophagy may overcome resistance to anti-cancer drugs, including anti-EGFR [12] and sorafenib [35] as well as butyrate.

The activation of AMPK by treating AICAR in BR colon cancer cells restored the inhibitory effect on cell proliferation through the inhibition of Akt/mTOR signaling and reduced the induction of autophagy, indicating that the activation of AMPK reduces resistance to butyrate and restores the sensitivity of butyrate relative to inhibitory effects on cell proliferation; thus, AMPK may play an important role in the regulation of resistance to butyrate by Akt/mTOR activation under stressful conditions. Similarly, stress and EGF induced Akt activation and promoted breast cancer progression, which developed drug resistance through AMPK-mediated Skp2 phosphorylation in this process [36].

ACC, a downstream target of AMPK, is an enzyme involved in the process of fatty acid synthesis from acetyl-CoA to malonyl-CoA [37]. During protective autophagy in BR colon cancer cells, phospho-ACC also decreased as phospho-AMPK decreased, possibly to effectively obtain energy for BR cell survival by inducing the accumulation of acetyl-CoA through a reduction in ACC levels. A decrease in phospho-ACC subsequently inhibited the enzymes involved in the fatty acid synthesis, such as ACLY and FASN, and may result in reduced production of fatty acids. This is in contrast to the results obtained for chemoresistant cancer cells. ACC expression increases in cetuximab-resistant head and neck squamous cell carcinoma to support cancer cells’ survival [38]. Overexpression of ACLY in HT29 colon cancer induced resistance to SN-38, an active metabolite of irrinothecan [39]. In addition, the overexpression of FASN in adriamycin-resistant MCF7 breast cancer cells (MCF7/AdVp3000) has been suggested as a new target for chemoresistance and seems to be a poor prognosis indicator for breast cancer patients [40].

However, as butyrate resistance is induced by chronic exposure to butyrate produced by digestion of dietary fiber, it can be viewed as a situation rich in butyrate as an energy source; thus, there is no need to catabolize fatty acid. Therefore, it is presumed that the expression of SCAD involved in β-oxidation of butyrate is considerably high with respect to obtaining energy from butyrate, and the expression of enzymes involved in fatty acid synthesis may be lower than those in PT cells since there is no need to induce lipogenesis due to its abundance in the BR colon cancer cells.

## 5. Conclusions

**Figure 8:**
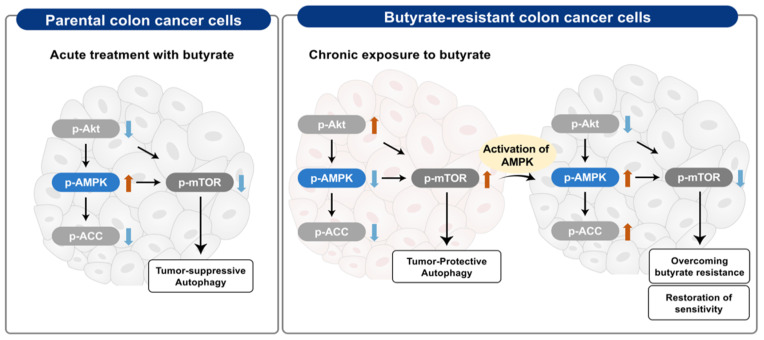
Summary of possible mechanisms in parental (PT) and butyrate-resistant (BR) colon cancer cells. p-, phospho-.

Taken together, a summary of this study is presented in Figure 8. In PT colon cancer cells, a single treatment with butyrate causes a decrease in phospho-Akt and results in an increase in phospho-AMPKα and a decrease in phospho-mTOR levels, inducing cancer-suppressing autophagy and anticancer effects. In BR colon cancer cells, phospho-Akt level increases and results in a decrease in phospho-AMPKα levels and an increase in phospho-mTOR levels, inducing autophagy that protects BR colon cancer cells through the suppression of fatty acid synthesis and developing resistance to butyrate. The activation of AMPK provides solutions for overcoming resistance and restores sensitivity to anti-cancer therapies as well as butyrate.

## Figures and Tables

**Table 1:** Forward and reverse primer sequences of *CPT1A*, *LCAD*, *SCAD*, *ACC*, *ACLY*, and *FASN* for quantitative real-time. reverse transcription PCR.

Gene	Forward Primer (5′→3′)	Reverse Primer (5′→3′)
*CPT1A*	ATCAATCGGACTCTGGAAACGG	TCAGGGAGTAGCGCATGGT
*LCAD*	TGCAATAGCAATGACAGAGCC	CGCAACTACAATCACAACATCAC
*SCAD*	CGGCAGTTACACACCATCTAC	GCAATGGGAAACAACTCCTTCTC
*ACC*	CTCCTGCTCATCACAGTATG	GCAAGGCTACTAAGGCAGG
*ACLY*	TCCAGGAGTCAAAATGATTGTG	ATCTCTCCAAGCTCATCAAAGC
*FASN*	CTTCCGAGATTCCATCCTACGC	TGGCAGTCAGGCTACACAAACG
*GAPDH*	TTCGACAGTCAGCCGCATCTTCT	AGGCGCCCAATACGACCAAATC

*CPT1A*, carnitine palmitoyltransferase 1A; *LCAD*, long-chain acyl-CoA dehydrogenase; *SCAD*, short-chain acyl-CoA de. hydrogenase; *ACC*, acetyl-CoA carboxylase; *ACLY*, ATP citrate lyase; *FASN*, fatty acid synthase.

**Table 2:** Mean IC_50_ ± standard deviation (*n* = 3) of sodium butyrate, oxaliplatin, doxorubicin, and 5-fluorouracil in PT and BR human colon cancer cells of HCT116, HT29, and SW480.

Drugs	HCT116		HT29		SW480	
	PT	BR	PT	BR	PT	BR
Sodium butyrate (mM)	4.91 ± 1.39	74.5 ± 4.36 ***	2.39 ± 0.146	33.5 ± 13.3 ***	4.01 ± 0.481	24.4 ± 0.805 ***
Oxaliplatin (µM)	3.83 ± 0.591	20.0 ± 0.679 ***	11.4 ± 2.72	54.2 ± 0.905 **	5.09 ± 1.94	40.7 ± 0.418 ***
Doxorubicin (nM)	44.3 ± 5.83	151 ± 36.8 **	296 ± 0.283	1036 ± 3.54 **	36.8 ± 8.21	170 ± 4.78 ***
5-Fluorouracil (µM)	4.43 ± 0.934	15.1 ± 0.721 ***	14.3 ± 1.27	52.0 ± 6.24 ***	4.78 ± 0.865	13.9 ± 1.28 ***

IC_50_, the concentration inhibiting the cell growth by 50%; PT, parental; BR, butyrate-resistant; **, *p* < 0.01; ***, *p* < 0.001.

## Data Availability

All data described in the study can be found in the article, and we do not have any supporting data.
